# Effect of methylglyoxal on the alteration in structure and digestibility of α‐lactalbumin, and the formation of advanced glycation end products under simulated thermal processing

**DOI:** 10.1002/fsn3.2211

**Published:** 2021-02-28

**Authors:** Yuekun Wu, Lu Dong, Yajing Wu, Dongyan Wu, Yan Zhang, Shuo Wang

**Affiliations:** ^1^ Tianjin Key Laboratory of Food Science and Health School of Medicine Nankai University Tianjin China; ^2^ State Key Laboratory of Food Nutrition and Safety Tianjin University of Science and Technology Tianjin China

**Keywords:** digestibility, methylglyoxal, protein‐bound advanced glycation end products, structural modification, thermal processing, α‐Lactalbumin

## Abstract

α‐Dicarbonyl compounds (α‐DCs) are a class of compounds generated during the thermal processing of food. Due to the high reactivity, α‐DCs were endowed with the ability to react with food components thus lowering nutrition value and even leading to a potential risk for food safety. In this study, methylglyoxal (MG), the most abundant α‐DCs, was selected to investigate the alteration effects on the structure and digestibility of α‐lactalbumin (αLA) under thermal processing (60–100°C). The results showed that the modification degree of αLA by MG increased with the rise of processing temperature, accompanied by the significant changes in molecular weight, intrinsic fluorescence, and secondary structures of αLA. High‐resolution mass spectrometry analysis identified that lysine (Lys) and arginine (Arg) are the modification sites, and N^ε^‐(carboxyethyl)‐L‐lysine is the main modification type. Since the Lys and Arg are also the cleavage sites of trypsin, the digestibility of MG modified αLA (MG‐αLA) by trypsin correspondingly decreased with an increase of processing temperature. The reacted Lys and Arg residues, and the protein‐bound AGEs were quantified, and the contents were found to be highly dependent on the temperature.

## Graphical abstract

Methylglyoxal (MG) can modify α‐lactalbumin (αLA) during the thermal processing of food (60–100°C). With the increase of temperature, the higher modification degree of αLA by MG, the more changes in spatial conformation and secondary structure of MG‐αLA. Modification sites and modification types of MG‐αLA were identified by HPLC‐MS/MS. The in vitro digestibility of MG‐αLA also decreased significantly with increasing temperature, accompanied by the more formation of protein‐bound advanced glycation end products.

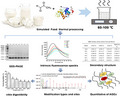

## 1 INTRODUCTION

Thermal processing is widely used in food industry, where a series of complex reactions occur between the food components, including the Maillard reaction, which was a typical reaction that contributes to the color and flavor of foods, but while generate some harmful substances, such as α‐dicarbonyl compounds (α‐DCs), acrylamide, furan, and advanced glycation end products (AGEs), which bring about potential risks to food safety and human health (Almajwal et al., 2020; Zhu et al., 2020).

α‐DCs are a class of active carbonyl compounds produced by Maillard reaction during food processing (Papetti et al., 2013), and more than 18 kinds of α‐DCs have been identified in various foods, among which methylglyoxal (MG) is one of the most representative (Arena et al., 2011; Marceau & Yaylayan, 2009). Since the activity of α‐DCs is much greater than that of glucose, MG can quickly react with protein amino acid residues during the thermal processing of food, affecting protein structures and functional properties. Under simulated thermal processing conditions (100–180°C for 15 min), MG significantly reduced the surface hydrophobicity and the digestibility of glutenin (Wang et al., 2019). After β‐lactoglobulin and β‐casein are, respectively, incubated with MG at 95°C for 1 hr, their digestibility was significantly decreased (Zhao et al., 2017). Similarly, after MG and myofibrillar proteins were incubated at 80°C for 48 hr, the water‐holding capacity and trypsin–chymotrypsin digestibility of the proteins were decreased as well (Luna & Estévez, 2019).

In addition, it has been confirmed that α‐DCs are the most important precursors of AGEs in Maillard reaction system (Cha et al., 2019). Dong et al. (2019) reported that the protein‐bound AGEs were formed in the reaction of MG and β‐lactoglobulin during the simulate dairy reheating. The AGEs formed in processed foods are one of significant factors affecting AGEs levels in human body (Rabbani & Thornalley, 2008), which are closely related to various chronic diseases, such as Alzheimer's, diabetes (Wang et al., 2012), kidney disease (Poulsen et al., 2013), cardiovascular (Uribarri et al., 2010), and cerebrovascular diseases (Losso et al., 2011). However, AGEs are a class of complex and heterogeneous compounds formed by reactions between carbonyl compounds and amino groups under nonenzymatic conditions (Wei et al., 2009). Due to the complexity and diversity of AGEs, the study about the mechanism of AGEs formation in specific food systems deserves to be explored, which is of great significance for evaluating the nutrition and safety of thermal processed foods.

α‐Lactalbumin (αLA), a globular protein with low molecular weight (~14.2 kDa), is one of two major whey proteins (Kinsella & Whitehead, 1989). It is widely used due to its high nutritional value and multi‐functional properties in various thermal processed foods, such as dairy beverages, infant formula, and nutritional bars (Arena et al., 2017; Carter & Drake, 2018; Foegeding et al., 2002; McGuffey et al., 2005). Since the wide existence of αLA, the structure and property changes of αLA during food thermal processing are critical to the nutrition and safety of food. At present, there is no relevant report about the relationship between the structure and property changes of αLA induced by MG and the formation of AGEs under the thermal processing of food.

In this study, a chemical model contained αLA and MG was carried out to investigate the effects of MG on the structure αLA under simulate thermal processing, including the changes in molecular weight, intrinsic fluorescence, and secondary structure, especially the analysis on the modification types and modification sites occurred in the primary structure of αLA. In addition, the in vitro gastrointestinal digestibility of MG modified αLA (MG‐αLA) and the formation of protein‐bound AGEs were also studied. These results will provide fundamental relevant information to the safety and nutritional of dairy products.

## 2 MATERIALS AND METHODS

### 2.1 Materials

Methylglyoxal (40% aqueous solution, w/w), αLA (L5385, calcium‐saturated, ≥85%, identified by SDS‐PAGE), trypsin (10,000 units/mg protein), pepsin (3,800 units/mg protein), and chymotrypsin (40 units/mg protein) were obtained from Sigma‐Aldrich, and N^ε^‐(carboxymethyl)‐L‐lysine (CML) and N^ε^‐(carboxyethyl)‐L‐lysine (CEL) were obtained from Toronto Research Chemicals. All of other reagents were analytical reagent grade. Ultrapure water was prepared using a Milli‐Q water purification system.

### 2.2 Simulated thermal processing on MG and αLA

Methylglyoxal and αLA were added in 10 mmol/L phosphate‐buffered solution (pH 6.8), and their final concentrations were 100 μmol/L and 1.5 mmol/L, respectively. The mixed solution was transferred into a capped glass bottle and heated, respectively, at 60, 70, 80, 90, and 100°C for 1 hr in a block heater (SBH200D/3; Stuart). Then, the reaction products were dialyzed at 4°C for 48 hr to remove the excess MG and lyophilized for the following analysis. The unheated αLA (UαLA) without MG was served as control.

### 2.3 Sodium dodecyl sulfate‐polyacrylamide gel electrophoresis (SDS‐PAGE)

Unheated α‐lactalbumin and MG‐αLAs were, respectively, analyzed by SDS‐PAGE with 3% stacking gel and 12% separating gel (Luna & Estévez, 2019). The samples (4 mg/ml of protein) were mixed with the loading buffer, and β‐mercaptoethanol at a volume ratio of 5:4:1, and boiled for 5 min. Then, 10 μl of each protein sample was loaded onto the gel. The electrophoresis was carried out at 80 V constant voltage. The gel was dyed with Coomassie Brilliant Blue R‐250, then discolored, and photographed.

### 2.4 Intrinsic fluorescence analysis

Unheated α‐lactalbumin and MG‐αLAs were, respectively, added in 10 mmol/L phosphate‐buffered solution (pH 7.0) to an ultimate concentration of 0.1 mg/ml. The intrinsic fluorescence of sample solutions was measured using a fluorescence spectrophotometer (Lumina, Thermo Fisher) at an excitation wavelength (Ex) of 280 nm and an emission wavelength (Em) of 300–400 nm.

### 2.5 Secondary structure analysis

Unheated α‐lactalbumin and MG‐αLAs were, respectively, added in 10 mmol/L phosphate‐buffered solution (pH 7.0) to an ultimate concentration of 2.0 mg/ml. The spectrum of MG‐αLA solution was measured using a Jasco‐815 Circular Dichroism (CD) Spectrometer (JASCO) with a wavelength range of 190–260 nm (Zhang et al., 2014). CDNN (http://gerald‐boehm.de/download/cdnn) was used to analyze the secondary structures of samples.

### 2.6 High‐resolution mass spectrometry analysis

The modification sites and types were identified by high‐resolution mass spectrometry according to a previously described method (Dong et al., 2019). The target bands of samples were cut from the SDS‐PAGE gels and decolorized by destaining solution. The dithiothreitol (10 mmol/L) and iodoacetamide (55 mmol/L) were used to reduce disulfide bridges of each sample, and then, the samples were digested by trypsin with an enzyme to protein ratio of 1:50 (w/w) for 12 hr at 37°C. The digested peptide solution was desalted with C18 tips, and the eluate was collected. The desalted peptides were separated using a C18 trap column (5 μm, 150 μm × 3 cm, 120 Å) and a C18 separation column (3 μm, 75 mm × 15 cm, 100 Å) on an Easy‐nLC1200 system (Thermo Fisher). Solvent A was 0.1% formic acid in water (v/v), and solvent B was 0.1% formic acid in acetonitrile (v/v). The flow rate was 600 nl/min, and the gradient elution program was applied (gradient: 0–6 min, 5%–10% solvent B; 10–43 min, 10%–30% solvent B; 43–53 min, 30%–45% solvent B; 53–54 min, 45%–95% solvent B; 54–60 min, 95% solvent B). The Orbitrap Fusion Lumos mass spectrometer (Thermo Fisher) was used to the analysis of proteolytic peptides. The primary resolution was set to 120,000 (*m/z* 200), and the secondary resolution was 30,000 (*m/z* 200). The parent ion scan range was *m/z* 350–1,550, and the daughter ion scan range was starting from *m/z* 120, followed by selecting the 20 most intense ions for secondary fragmentation. High energy capture dissociation (HCD) fragmentation mode was used to acquire the fragment ions of each peptide.

### 2.7 The in vitro gastrointestinal digestibility of MG‐αLAs

The in vitro gastrointestinal digestibility of MG‐αLAs was determined according to the reported method (Wen et al., 2019). Briefly, 5 mg of MG‐αLA was added to 3.5 ml of simulated gastric fluid (the concentration of pepsin was 182 U/mg of protein) and then digested at 37°C for 1 hr. The sample solution was adjusted to pH 7.0 with NaHCO_3_ (1 mol/L) to inactivate the pepsin. Simulated intestinal fluid containing chymotrypsin (0.5 U/mg of protein) and trypsin (40 U/mg of protein) was added to the sample solution and digested for 2 hr, then using a boiling water bath to stop the reaction. After vitro digestion, the product was diluted with o‐phthalaldehyde. The fluorescence emission of the sample solution was measured using a plate reader (Varioskan LUX, Thermo Scientific) with an Ex of 340 nm and an Em of 450 nm. The standard curve was established with different concentrations of tryptophan (0.01, 0.05, 0.1, 0.2, 0.5, and 0.6 mmol/ml), and the degree of protein hydrolysis (DH) was calculated:

$$\text{DH}\left(\%\right)=\frac{h_{t}}{h_{p}}\times 100$$

where *h*_t_ is the millimoles of free amine groups per gram of protein in the test sample, and *h*_p_ is the millimoles of free amino groups per gram of protein that are completely hydrolyzed (8.6 mmol/g for αLA) (Deng et al., 2018).

### 2.8 Determination of reacted Lys and Arg

The content of free Lys in MG‐αLA was measured according to the reference (Ashraf et al., 2015). The standard curve of the correlation between the concentration of Nα‐(acetyl)lysine (0–1.5 mmol/L) and the fluorescence intensity was used to calculate the concentration of free Lys residues.

The content of free Arg in MG‐αLA was determined with 9,10‐phenanthraquinone assay (Ashraf et al., 2015). The standard curve of the correlation between the concentration of Nα‐(acetyl)arginine (0–0.5 mmol/L) and the fluorescence intensity was used to calculate the concentration of free Arg. The percentage of reacted Arg (or Lys) in MG‐αLA was calculated:

$$\text{The}\;\text{ratio}\;\text{of}\;\text{reacted}\;\text{Lys}/\text{Arg}\left(\%\right)=\frac{C_{u}‐C_{t}}{C_{u}}\times 100$$

where *C*_u_ and *C*_t_ are the content of free Arg (Lys) of UαLA and MG‐αLA, respectively.

### 2.9 Determination of protein‐bound CML and CEL

The amount of protein‐bound CML and CEL in MG‐αLAs was determined according to the reported method (Teerlink et al., 2004). Briefly, 4 mg of MG‐αLAs was dissolved with 1.5 ml of borate buffer (0.2 mol/L, pH 9.2) and 1 ml of sodium borohydride (2 mol/L) to standing at 4°C for 8 hr, and then, 2.5 ml of 12 mol/L HCl was added and the hydrolysis was carried out at 110°C for 24 hr. After that, the hydrolyzed samples were purified by solid phase extraction with an Agela PCX column (200 mg/3 ml, Agela). Then, the eluates containing CML and CEL were evaporated to dryness and the residue was reconstituted in Milli‐Q water–methanol (80:20, v/v). The content of protein‐bound CML and CEL was analyzed with liquid chromatography tandem mass spectrometry (LC–MS/MS, Agilent). The purified samples were separated using a Waters HILICA C18 column (2.1 mm × 150 mm, 4 μm particle size). 0.1% Formic acid in water (v/v) and methanol were used as solvent A and B, respectively. The flow rate was set as 0.2 ml/min, and the isocratic elution was performed 6 min. The column temperature was 35°C, and the injection volume was 2 μl. Mass spectroscopy was carried out in positive electrospray ionization mode and multiple reaction monitoring mode. The atomized gas pressure was set as 40 psi, capillary voltage as 4,000 V, and dryer temperature as 350°C. The product ions at *m/z* 83.9 and 129.8 (CML) and at *m/z* 84.3 and 130.2 (CEL) were used for quantification and confirmation, respectively.

### 2.10 Statistical analysis

All the experiments except for the mass spectrometry data were performed in three parallel experiments, and the results are expressed as the mean ± standard deviation. Data were evaluated by one‐way analysis of variance (ANOVA), and the significance level was established using Duncan's post hoc test at *p* < 0.05. The mass spectrometry data were retrieved by Protein Metrics Inc (PMI) Byonic and database retrieval parameters which were set according to references which were shown in Tables S1 and S2 (Arena et al., 2014; Henle, 2005; Mittelmaier & Pischetsrieder, 2011; Renzone et al., 2015; Tu et al., 2017).

## 3 RESULTS AND DISCUSSION

Methylglyoxal and αLA were incubated and reacted under the simulated common processing temperatures (60–100°C) to prepare the MG‐αLAs; then, the structure alteration of MG‐αLAs was analyzed by molecular weight, intrinsic fluorescence, and secondary structure. The modification sites of MG‐αLAs were identified by high‐resolution mass spectrometry. In addition, the in vitro gastrointestinal digestibility of MG‐αLAs, the reacted ratio of Lys and Arg, and the formation of protein‐bound AGEs were analyzed to explore the negative impacts of MG modification during the thermal processing of food.

### 3.1 Changes in molecular weight of MG‐αLAs

**FIGURE 1:**
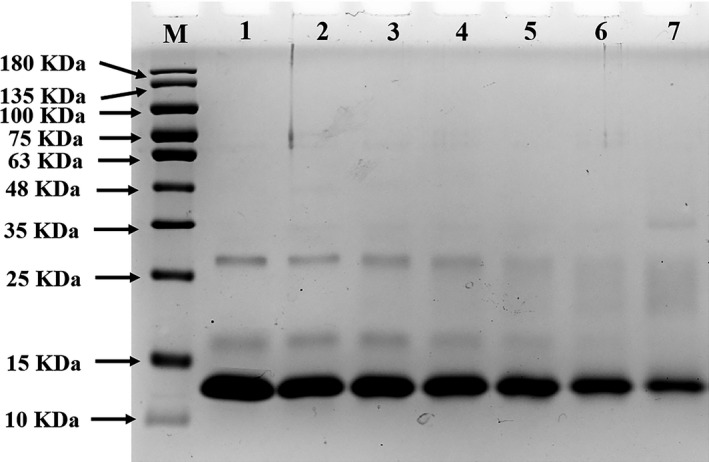
SDS‐PAGE of UαLA and MG‐αLAs (M, Marker proteins; Lane 1, UαLA; Lane 2, UαLA treated at 100°C; Lane 3–7, MG‐αLAs at 60–100°C)

Sodium dodecyl sulfate‐polyacrylamide gel electrophoresis was used to monitor the molecular weight changes of MG‐αLAs (Figure 1). An obvious band at about 14 kDa appeared in UαLA (lane 1), which is consistent with the molecular weight of αLA (14.2 kDa). After heated at 100°C without MG, the changes in the bands of αLA were not obvious (lane 2). After heated with MG, the bands of αLA significantly faded, and new dispersed bands were observed at around 14~28 kDa and became wider gradually upon the increasing temperature (60–100°C, lane 3–7). The above results indicated that the modification of αLA by MG occurred and the modification degree was positively correlated with reaction temperature. The maximum molecular weight of new dispersed band was about twice as large as the molecular weight of αLA, and this might be due to the fact that MG has two active carbonyl groups, which endows it to be a linker to induce the covalent interactions between protein molecules, leading to the formation of dimers (Anna & Davies, 2019). These results were similar with the previous research work that MG modified β‐LG to a certain degree, further forming β‐LG‐related derivatives with different molecular weights (Dong et al., 2019).

### 3.2 Intrinsic fluorescence analysis

**FIGURE 2:**
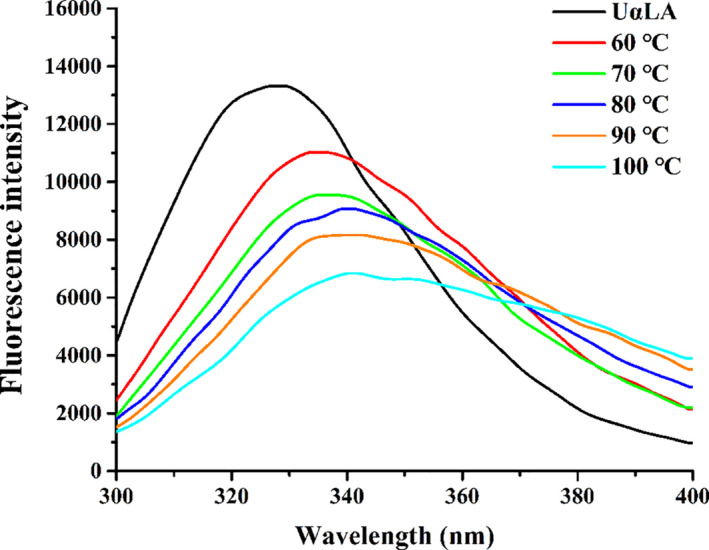
Intrinsic fluorescence spectra of UαLA and MG‐αLAs

The intrinsic fluorescence spectrum was closely related to the polarity of molecular environments around aromatic amino acid residues (e.g., tryptophan (Trp), tyrosine (Tyr), and phenylalanine (Phe)) and can reflect the conformation changes of proteins (Luna & Estévez, 2018). The intrinsic fluorescence spectra of UαLA and all MG‐αLAs at different temperatures were shown in Figure 2. The UαLA has a strong fluorescence emission peak at 328 nm after being excited at 280 nm, but it decreased dramatically after reacted with MG under different temperature (60–100°C), with the fluorescence intensity reduced from 13,311.8 to 6,839.7, and *λ*_max_ shifted from 328 to 341 nm. The one kind of explanation was that more Trp or Tys residues were exposed to solvent after αLAs were modified by MG, which could lead to the quenching of fluorescence (Mir et al., 2014). Another explanation was that more Trp or Tys residues were buried within αLAs because of shielding effect after the reacted with MG. These can result in a reducing of fluorescence intensity of proteins. In addition, the red shift of *λ*_max_ also indicates that Trp or Tys residues are exposed to a more polar environment (Mir et al., 2014). Therefore, the different fluorescence intensities of MG‐αLAs reflect the different degrees of conformational changes of MG‐αLAs, indicating that MG modification has significant effect on the spatial conformation of αLA and higher processing temperature can increase the degree of changes in protein conformation.

### 3.3 Secondary structure analysis

According to the changes in spatial conformation of MG‐αLAs, the secondary structure changes were further analyzed by CD spectroscopy (Table 1). Compared with UαLA, the α‐helix and random coil ratio of MG‐αLAs, respectively, decreased from 22.60% to 18.60% and from 32.97% to 30.20%, while the β‐sheet ratio gradually increased from 19.57% to 25.20% (60–100°C). Bakhti et al. (2007) found that the increase in content of β‐sheet is likely to promote the intermolecular interactions between proteins to form aggregates, furthering making the protein conformation more stable. This suggested that MG‐αLA was partially unfolded and reorganized to be a more stable structure. Yang et al. (2019) also found similar changes in secondary structures on the investigation of conformational alteration in ovalbumin induced by glycation with different monoses. SDS‐PAGE analysis revealed the formation of new dimers, and intrinsic fluorescence analysis also showed the changes in spatial conformation of MG‐αLAs. The changes in secondary structures further proved that MG modification can alter the structure of αLA, and as the temperature increases, the modification degree increases.

**TABLE 1:** Secondary structural contents of UαLA and MG‐αLAs

Samples	α‐helix (%)	β‐sheet (%)	β‐turn (%)	Random coil (%)
UαLA	24.80 ± 3.21^a^	18.00 ± 2.25^c^	19.20 ± 3.20^a^	33.80 ± 1.13^a^
60°C	22.60 ± 0.70^ab^	19.57 ± 1.11^bc^	17.50 ± 0.20^a^	32.97 ± 0.21^a^
70°C	21.80 ± 0.20^b^	20.07 ± 0.46^bc^	18.07 ± 0.38^a^	31.60 ± 0.17^b^
80°C	20.23 ± 0.58^bc^	21.90 ± 1.22^b^	18.50 ± 1.17^a^	30.70 ± 0.20^c^
90°C	18.93 ± 0.55^c^	24.63 ± 1.67^a^	19.00 ± 0.20^a^	30.60 ± 0.26^c^
100°C	18.60 ± 0.36^c^	25.20 ± 1.23^a^	19.23 ± 0.15^a^	30.20 ± 0.17^c^

### 3.4 Identification of modification sites and modification types of MG‐αLAs

**FIGURE 3:**
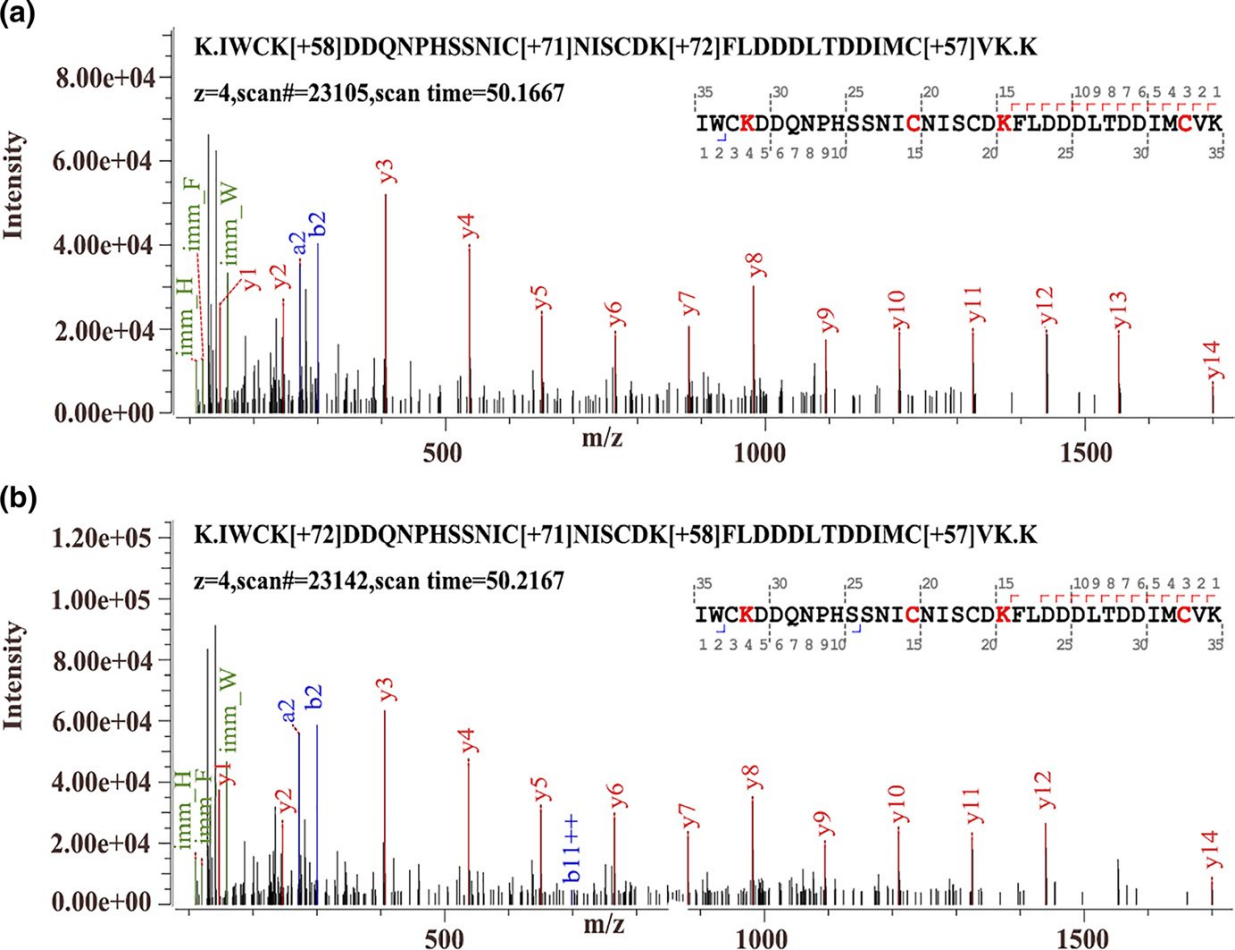
Secondary mass spectrometry spectra of the (a) modified peptide 78–113 (CML and CEL), and (b) modified peptide 78–113 (CEL and CML) of MG‐αLAs

In order to reveal the changes in primary structure of MG‐αLAs, the modified sites and modification types were identified by high‐resolution mass spectrometry and the results of modification types and sites are showed in Table 2. The number of modification types produced by MG modification on αLA at different temperatures was not all identical, but mainly distributed on Lys and Arg. The example peptides of IWCkDDQNPHSSNICNISCDkFLDDDLTDDIMCVK, with different modification types (CML and CEL), were showed in Figure 3. There are four kinds of modified types on Lys including CML, CEL, pyrraline, and Pyr‐CML and five modified types on Arg including MG‐H1, MG‐DH, Argpyrimidine, THP, and DHP in MG‐αLAs under different heating temperatures.

**TABLE 2:** Modified types and sites of MG‐αLA (60°C, 80°C)

Sequence	+Da	Modification types and modification sites	
		60°C	80°C
CEVF**R**ELKDLK	54	MG‐H1 (R29)	MG‐H1 (R29)
CEVF**R**ELKDLK	72	MG‐DH (R29)	‐
CEVF**R**ELKDLK	80	Argpyrimidine (R29)	Argpyrimidine (R29)
CEVF**R**ELKDLK	144	THP (R29)	THP (R29)
CEVF**R**ELKDLK	126	DHP (R29)	DHP (R29)
CEVFREL**K**DLK	72	CEL (K32)	CEL (K32)
CEVFREL**K**DLK	108	Pyrraline (K32)	‐
IWC**K**DDQNPHSSNICNISCDK	58	CML (K81)	CML (K81)
IWC**K**DDQNPHSSNICNISCDK	72	CEL (K81)	‐
IWCKDDQNPHSSNICNISCD**K**	40	Pyr‐CML (K98)	‐
IWCKDDQNPHSSNICNISCD**K**	58	CML (K98)	CML (K98)
IWCKDDQNPHSSNICNISCD**K**	72	CEL (K98)	CEL (K98)
FLDDDLTDDIMCV**K**K	40	Pyr‐CML (K112)	Pyr‐CML (K112)
FLDDDLTDDIMCV**K**K	72	CEL (K112)	CEL (K112)

Note

“‐” Indicates “not detected.”

CEL—the main modification type of Lys, was found at K32, K81, K98, and K112 in MG‐αLA at 60°C and was found at K32, K98, and K112 at 80°C. CML was detected at K81 and K98, and Pyr‐CML was found at K112 in MG‐αLAs at 60 and 80°C. Pyrraline was found at K32 in MG‐αLA at 60°C. MG‐H1 and DHP, Argpyrimidine, and THP were detected at R29 at 60 and 80°C. MG‐DH was only detected at 60°C as an Arg‐modified type in MG‐αLAs. It is worth noting that there are CML, CEL, and pyrraline modification at 60°C on site K32 where CML and CEL modification exist at 80°C. The same situation occurs at the K98 site as well, which mean that MG‐αLA will produce more CML and CEL at 80°C. These results were consistent with changes in secondary structure of MG‐αLAs, and both indicated that αLA can be modified by MG, and the modification degree increases with the increase in reheating temperature.

### 3.5 The changes in digestibility of MG‐αLAs

**FIGURE 4:**
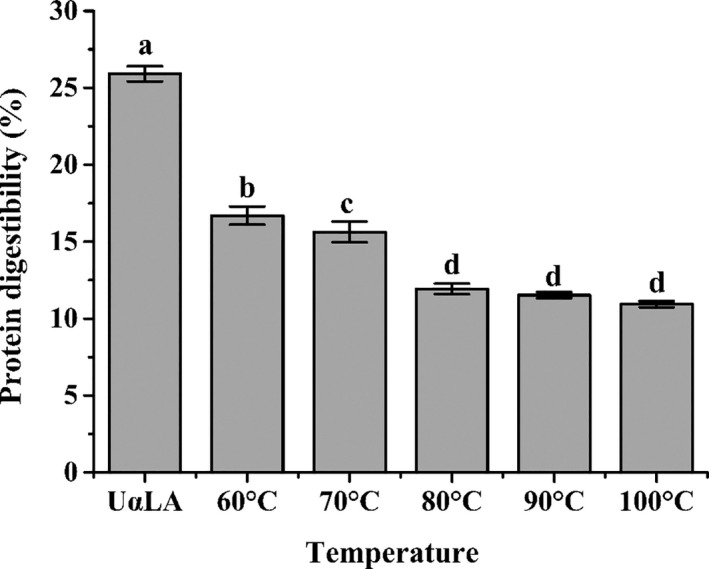
The digestibility of UαLA and MG‐αLAs

Heat treatment can induce reactions between various components of food, which might lead to the changes in digestibility of food components (Teerlink et al., 2004). The in vitro gastrointestinal digestibility results of MG‐αLAs were shown in Figure 4. The digestibility values of UαLA and MG‐αLAs (60–100°C) were 25.91%, 16.71%, 15.64%, 11.93%, 11.53%, and 10.93%, respectively. All the digestibilities of MG‐αLAs (60–100°C) were lower than UαLA, which indicated that MG modification can significantly change the digestibility of αLA. The high‐resolution mass spectrometry analysis demonstrated that the modification sites of MG on αLA were Lys and Arg, which also correspond to the cleavage sites of trypsin. Furthermore, MG can induce the aggregation of protein, leading to the masking of protease cleavage sites (Wang et al., 2019). Therefore, the reduced digestibility of MG‐αLAs is the result of structural changes in MG‐αLAs. Considering that the digestion and absorption of protein is important for human health, the decrease in protein digestibility means loss of protein nutrition and may have a negative effect on the human body.

### 3.6 Quantitation of reacted Lys and Arg residues of MG‐αLAs

The reacted percentage of Arg and Lys in MG‐αLAs were determined to further verify the alteration degree of the protein structures and the nutritional loss in foods. As shown in Table 3, when the temperature ranged from 60 to 100°C, the percentage of reacted Lys in MG‐αLAs increased from 6.55% to 73.77%, and the percentage of reacted Arg was 100% in all MG‐αLAs. The results indicated that Lys and Arg participated in the modification reaction, further verified the results of modification sites in MG‐αLAs identified by LC‐MS/MS. With the increase in temperature, the reacted Lys content of MG‐αLAs was increased, indicating the increased modification degree on the structure of αLA and the reaction temperature led to the significantly reducing of free Lys residues. There is only one Arg residue on the amino acid sequence of αLA, which could be easily reacted and consumed in the reaction; therefore, the percentage of reacted Arg reached to 100% under all the processed temperature (60–100°C). Arena et al. (2014) also found that the modification of protein causes the loss of amino acids in dairy products. In our daily diet, Lys is an essential amino acid in human body, Arg is also an essential amino acid to baby, therefore, the higher temperature is performed, the more loss of essential amino acid in MG‐αLAs, meaning the lower nutritional value of foods containing αLAs.

**TABLE 3:** The reacted percentage of Lys and Arg in MG‐αLAs

Samples	Reacted ratio of Lys (%)	Reacted ratio of Arg (%)
60°C	6.55	100
70°C	13.93	100
80°C	20.49	100
90°C	33.61	100
100°C	73.77	100

### 3.7 Formation of protein‐bound CML and CEL in MG‐αLAs

The protein‐bound CML and CEL were quantified during the thermal process (60–100°C), and their contents significantly increased with the raising temperature (Table 4). The content of protein‐bound CEL increased from 28.92 to 118.20 mg/kg protein with the temperature increasing from 60 to 100°C, and the protein‐bound CML content also showed a similar upward trend from 34.00 to 165.58 mg/kg protein. The contents of protein‐bound CML and CEL generated at 100°C were both approximately five times higher than those at 60°C. It indicated that high temperature acts a major role in promoting the formation of protein‐bound AGEs. In addition, the results of high‐resolution mass spectrometry found that the AGEs were generated by the modification of αLA by MG, which means that at common thermal processing conditions, MG can not only change the structure and digestibility of αLA, but also promote the harmful protein‐bound AGEs. Hence, higher food processing temperatures not only bring attractive color and aroma, but also cause harmful changes in protein‐rich food.

**TABLE 4:** The contents of CML and CEL in UαLA and MG‐αLAs

Samples	CML (mg/kg protein)	CEL (mg/kg protein)
UαLA	‐	‐
60°C	34.00 ± 1.09^e^	28.92 ± 0.31^e^
70°C	58.19 ± 1.77^d^	58.47 ± 1.29^d^
80°C	73.63 ± 3.98^c^	76.12 ± 0.68^c^
90°C	112.23 ± 2.25^b^	94.28 ± 3.18^b^
100°C	165.58 ± 3.15^a^	118.20 ± 2.97^a^

Note

“‐” indicates the value was below the limit of detection.

## 4 CONCLUSIONS

Under simulated thermal processing (60–100°C), MG can modify the structure of αLA, leading to the significant changes in molecular weight, intrinsic fluorescence, and secondary structures. The modification sites of MG‐αLAs were found at Lys and Arg residues—the cleavage sites of trypsin, thus leading to a decrease in the digestibility of MG‐αLAs. The ratio of reacted Lys and Arg and the formation contents of protein‐bound CML and CEL also increased with the rising temperature. These results suggested that choosing an appropriate processing temperature or avoiding unnecessary heating is conducive to reducing the loss of nutrients and the generation of harmful compounds in dairy products.

## CONFLICT OF INTEREST

The authors declare that they have no conflicts of interest.

## ETHICAL APPROVAL

This study does not involve any human or animal testing.

## Supporting information

Tables S1 and S2

## Data Availability

Research data are not shared.
