# Supplementary material for: Effect of methylglyoxal on the alteration in structure and digestibility of α‐lactalbumin, and the formation of advanced glycation end products under simulated thermal processing
Source: Food Sci Nutr. 2021 Feb 28;9(4):2299–307. doi: 10.1002/fsn3.2211 (PMC8020911; doi:10.1002/fsn3.2211)
Supplement: Supplementary file 1 — Tables S1 and S2 [file FSN3-9-2299-s001.docx]

**Supplementary Material for:**

Effect of methylglyoxal on the alteration in structure and digestibility of α-lactalbumin, and the formation of advanced glycation end products under simulated thermal processing

**Authors:** Yuekun Wu, Lu Dong, Yajing Wu, Dongyan Wu, Yan Zhang, Shuo Wang

| **Table S1** Database retrieval parameters | |
| --- | --- |
| Item | Value |
| Enzyme | Trypsin (full) |
| Max Missed Cleavages | 2 |
| Peptide Mass Tolerance | ± 10 ppm |
| Fragment Mass Tolerance | ± 20 ppm |
| Peptide/ Protein False Discovery Rate (FDR) | Auto Cut, 0-5% |

| **Table S2** Variable modifications was set in analysis of modified peptides | |
| --- | --- |
| Variable modifications | Mass shift |
| Carbamidomethyl | C/+57.0214 |
| Oxidation | M/+15.9949 |
| Deamidated | N, Q/+0.9840 |
| Propionamide | C/+71.0371 |
| MG-derived Dihydroxyimidazolines (MG-DH) | R/+72.0205 |
| Pyrraline-carboxymethyllysine (Pyr-CML) | K/+40.0307 |
| methylglyoxal-derived hydroimidazolone 1 (MG-H1) | R/+54.0100 |
| N^ε^-(carboxymethyl) lysine (CML) | K/+58.0049 |
| N^ε^-(carboxyethyl) lysine (CEL) | K/+72.0205 |
| Arg-pyrimidine (Arg-P) | R/+80.0256 |
| Pyrraline (Pyr) | K/+108.0205 |
| Dihydropyrimidine (DHP) | R/+126.0311 |
| Tetra-hydropyrimidine (THP) | R/+144.0417 |
